# Perivascular Neuropilin‐1 expression is an independent marker of improved survival in renal cell carcinoma

**DOI:** 10.1002/path.5380

**Published:** 2020-01-29

**Authors:** Eric Morin, Cecilia Lindskog, Martin Johansson, Lars Egevad, Per Sandström, Ulrika Harmenberg, Lena Claesson‐Welsh, Elin Sjöberg

**Affiliations:** ^1^ Department of Immunology, Genetics and Pathology Uppsala University Uppsala Sweden; ^2^ Department of Laboratory Medicine Lund University Lund Sweden; ^3^ Department of Oncology‐Pathology Karolinska Institutet Stockholm Sweden

**Keywords:** Neuropilin, NRP1, VEGF, VEGFR2, *trans*‐complex, renal cell carcinoma (RCC), kidney cancer, *in situ* proximity ligation assay (PLA)

## Abstract

Renal cell carcinoma (RCC) treatment has improved in the last decade with the introduction of drugs targeting tumor angiogenesis. However, the 5‐year survival of metastatic disease is still only 10–15%. Here, we explored the prognostic significance of compartment‐specific expression of Neuropilin 1 (NRP1), a co‐receptor for vascular endothelial growth factor (VEGF). NRP1 expression was analyzed in RCC tumor vessels, in perivascular tumor cells, and generally in the tumor cell compartment. Moreover, complex formation between NRP1 and the main VEGF receptor, VEGFR2, was determined. Two RCC tissue microarrays were used; a discovery cohort consisting of 64 patients and a validation cohort of 314 patients. VEGFR2/NRP1 complex formation in *cis* (on the same cell) and *trans* (between cells) configurations was determined by *in situ* proximity ligation assay (PLA), and NRP1 protein expression in three compartments (endothelial cells, perivascular tumor cells, and general tumor cell expression) was determined by immunofluorescent staining. Expression of NRP1 in perivascular tumor cells was explored as a marker for RCC survival in the two RCC cohorts. Results were further validated using a publicly available gene expression dataset of clear cell RCC (ccRCC). We found that VEGFR2/NRP1 *trans* complexes were detected in 75% of the patient samples. The presence of *trans* VEGFR2/NRP1 complexes or perivascular NRP1 expression was associated with a reduced tumor vessel density and size. When exploring NRP1 as a biomarker for RCC prognosis, perivascular NRP1 and general tumor cell NRP1 protein expression correlated with improved survival in the two independent cohorts, and significant results were obtained also at the mRNA level using the publicly available ccRCC gene expression dataset. Only perivascular NRP1 expression remained significant in multivariable analysis. Our work shows that perivascular NRP1 expression is an independent marker of improved survival in RCC patients, and reduces tumor vascularization by forming complexes in *trans* with VEGFR2 in the tumor endothelium. © 2019 The Authors. *The Journal of Pathology* published by John Wiley & Sons Ltd on behalf of Pathological Society of Great Britain and Ireland.

## Introduction

Renal cell carcinoma (RCC) represents approximately 3% of all adult cancers worldwide, with an incidence of 337 860 new cases per year and 143 406 deaths in 2012 1. The incidence has been increasing since the 1990s but has leveled off in recent years 2, 3, 4, 5. Internationally, about 25% of patients with RCC have advanced disease (locally invasive or metastatic disease) at diagnosis, and for 30% the cancer recurs after resection 6. Despite the development of novel targeted therapies in recent years, RCC treatment is challenging once metastasis is manifest, resulting in a 5‐year survival of only around 10–15% 7, 8.

There are two main RCC tumor types; clear cell and papillary. Clear cell RCC (ccRCC) represents about 70% of all cases 9 and is characterized by inactivating mutations in the von Hippel–Lindau gene (*VHL*) 10, 11. *VHL* encodes the von Hippel–Lindau protein, which is critical in targeting the transcription factor hypoxia inducible factor (HIF) for degradation. In tumors with *VHL* mutations, HIF is constitutively stable, promoting the expression of a wide range of genes. These include vascular endothelial growth factor (VEGF), a potent inducer of tumor angiogenesis through binding to its receptor (VEGFR2) 12. In recent years, progress has been made in the treatment of RCC patients with advanced disease, to a large extent due to the introduction of anti‐angiogenic drugs targeting the VEGF pathway, including the kinase inhibitors sunitinib, pazopanib, axitinib, and cabozantinib 4, 13 and the anti‐VEGF antibody bevacizumab (in combination with interferon) 14. More recently, immune checkpoint inhibitors have been shown to improve overall survival and are now approved for treatment of advanced RCC 15.

Neuropilin 1 (NRP1) is a nonenzymatic transmembrane glycoprotein that binds VEGF to form a ternary complex with VEGFR2 on endothelial cells, potentiating downstream signaling to induce proliferation and migration 16, 17, 18. In addition, NRP1 is expressed by neuronal, epithelial, inflammatory, and tumor cells 19, 20. VEGFR2/NRP1 complexes can form in two configurations. When both molecules are expressed by the same cell, such as endothelial cells, complexes are formed in *cis*, while expression on adjacent cells results in formation of complexes in *trans*, between the cells 16.

We have shown previously that the interaction of tumor cell NRP1 with endothelial VEGFR2 in *trans* arrests the receptor on the cell surface, suppressing tumor angiogenesis and growth *in vivo* 21. We recently demonstrated that VEGFR2/NRP1 complexes are formed in human pancreatic ductal adenocarcinoma, and the presence of VEGFR2/NRP1 *trans* complexes was identified as an independent marker of improved overall survival 22. In this study, we explored whether high prevalence of VEGFR2/NRP1 complexes or the expression of NRP1 by perivascular tumor cells impact RCC patient prognosis.

## Materials and methods

### Ethical statement

All participating patients gave their written informed consent, and sample collection was made with the approval of the regional research ethics board of Stockholm (Dnr. 2010/1339 32) and Lund (Dnr 282/2007). The studies were performed in compliance with the 1975 Declaration of Helsinki, as revised in 1983.

### Patient material

Two tissue microarrays (TMAs) were used, referred to as the discovery and validation cohorts. The discovery cohort consists of tumor biopsies from 64 patients diagnosed with RCC between 1997 and 2005 at Karolinska University Hospital, Stockholm, Sweden. Tumors were evaluated histologically by a pathologist, and representative tumor parts were chosen when establishing the TMA. Each tumor is represented by two 1‐mm diameter core punch biopsies. All patients developed metastatic disease and were treated with sunitinib as first line treatment. Patients were classified according to the TNM classification system for malignant tumors. T stage describes tumor size and tissue invasion, N stage describes the spread to regional lymph nodes, and M stage describes metastatic spread to distant organs. M stage in this cohort describes the presence of metastasis at diagnosis 23.

The validation cohort consists of 314 RCC patients diagnosed between 1978 and 1996 at Skåne University Hospital Malmö, Sweden. The patients did not receive adjuvant therapy. Data for patients included sex, age, tumor stage, Fuhrman grade, metastasis present at diagnosis, pathological classification, and survival time. Primary tumor sections were revised by a certified pathologist, confirming the RCC diagnosis and selecting representative tumor areas of two 1‐mm diameter core biopsies to be included in the TMA. Data of patient characteristics, treatment, and overall survival were collected in clinical registries. Details about the cohorts are presented in Table 1. Both cohorts have been used in previous biomarker studies 24, 25.

**Table 1:** Association of perivascular NRP1 status with clinicopathological characteristics in the discovery and validation cohorts

	Discovery cohort				Validation cohort			
	Total	Negative	Positive	*p*‐value	Total	Negative	Positive	*p*‐value
	n=63	n=18	n=45		n=297	n=89	n=208	
**Sex**								
Female	14	6	8	0.197^1^	129	34	95	0.252^1^
Male	49	12	37		168	55	113	
**Age**								
<60	12	3	9	1^1^	98	27	71	0.59^1^
≥60	51	15	36		196	61	135	
Missing					3	1	2	
**Histology**								
Non clear cell	4	3	1	0.070^1^	21	12	9	0.005* ^,1^
Clear cell	58	15	43		236	62	174	
Missing	1	0	1		40	15	25	
**MSKCC score**								
Low	25	8	17	0.865^2^				
Intermediate	31	8	23					
High	3	1	2					
Missing	4	1	3					
**Fuhrman grade**								
1					113	15	98	<0.001* ^,1^
2					105	32	73	
3					54	28	26	
4					21	13	8	
Missing					4	1	3	
**T stage**								
1	10	3	7	0.935^2^	33	6	27	0.022* ^,1^
2	14	4	10		38	9	29	
3	37	11	26		34	10	24	
4	1	0	1		61	28	33	
Missing	1	0	1		131	36	95	
**M stage**								
0	39	10	29	0.573^1^	239	59	180	<0.001* ^,1^
1	24	8	16		58	30	28	

Statistical analysis: ^1^Fisherʼs exact test, ^2^Pearsonʼs chi‐square.

Abbreviations: M stage, presence of distant metastasis; MSKCC score, Memorial Sloan‐Kettering Cancer Center score; T stage, size or direct extent of the primary tumor.

* Denotes statistical difference (*p* < 0.05).

### Analyses of gene expression datasets

*NRP1* mRNA data from 12 cancer types and normal control tissues publicly available transcriptome data generated by The Cancer Genome Atlas (TCGA) Research Network (http://cancergenome.nih.gov/) and the genotype tissue expression (GTEx) project (https://www.gtexportal.org/) were analyzed. Data were accessed through the web‐based tool Gene Expression Profiling Interactive Analysis (GEPIA) (http://gepia.cancer-pku.cn) 26. Data are presented as transcripts per million. Cut‐off for significant difference between tumor expression and normal control was a log_2_ fold‐change of 1 and a *p*‐value <0.05. Differences between cancer types were not analyzed. The correlation between *NRP1* transcript levels and survival was analyzed in a ccRCC patient cohort with publicly available transcriptome data (KIRC), generated by TCGA (http://cancergenome.nih.gov/). The dataset consists of gene expression data from 534 patients with ccRCC. Dichotomization of the KIRC gene expression dataset was performed using the same cut‐off as in the RCC cohort, where the highest three quartiles were considered positive for *NRP1* expression.

### Antibodies and reagents

Anti‐VEGFR2 antibody (AF357; R&D Systems, Minneapolis, MI, USA) was used at a 1:100 dilution in an *in situ* proximity ligation assay (PLA). Anti‐VEGFR2 (2479; Cell Signaling Technology, Danvers, MA, USA) was used for immunofluorescent staining (IF) at a 1:150 dilution. Anti‐NRP1 antibody (60067–1; Proteintech, Rosemont, IL, USA) was used for PLA at a 1:100 dilution. Anti‐NRP1 (AF566; R&D Systems) at a 1:100 dilution was used for IF. Dylight‐650 conjugated anti‐CD34 (NBP2‐44567C; Novus Biologicals, Littleton, CO, USA) was used for *in situ* PLA at a 1:100 dilution. Anti‐CD34, clone QBEnd10 (IR63261‐2; Agilent Technologies, Santa Clara, CA, USA) was used for IF at a 1:100 dilution. Secondary antibodies, Alexa Fluor 488 donkey anti‐mouse, Alexa Fluor 647 donkey anti‐mouse, Alexa Fluor 555 donkey anti‐rabbit, Alexa Fluor 488 donkey anti‐goat (Invitrogen, Carlsbad, CA, USA), and Alexa Fluor 647 donkey anti‐goat (Jackson ImmunoResearch, Philadelphia, PA, USA) were used for IF at a 1:400 dilution, (see supplementary material, Table S1 for details). Isotype control antibodies used for IF were the following: goat IgG (0500–000‐003, Jackson ImmunoResearch), rabbit IgG (2729, Cell Signaling Technology), and mouse IgG1, kappa (554 121, BD Biosciences, San Jose, CA, USA).

### Immunofluorescence staining (IF)

Formalin‐fixed paraffin‐embedded (FFPE) TMA slides were deparaffinized using an EtOH gradient (xylene, 100% EtOH, 95% EtOH, 70% EtOH) followed by epitope retrieval using a microwave at 2 × 5 min in target retrieval buffer, pH 9 (Agilent Technologies). Slides were washed in Tris‐buffered saline (TBS) supplemented with 0.1% Tween 20, before blocking in 10% normal donkey serum supplemented with 1% bovine serum albumin (BSA) in TBS. Slides were incubated with primary antibodies overnight at 4 °C and with secondary antibodies for 1 hour at room temperature, thereafter with Hoechst 33342 before mounting with Fluoromount mounting medium (Merck, Kenilworth, NJ, USA). The specificity and sensitivity of antibodies against NRP1, VEGFR2, and CD34 were validated by staining of porcine aortic endothelial (PAE) cells stably overexpressing human VEGFR2 and NRP1 (as described in detail previously 21, 22) and RCC tumor tissue, with target specific antibodies or the corresponding isotype control.

### *In situ* proximity ligation assay (PLA)

Sections for *in situ* PLA were deparaffinized using xylene and then an EtOH gradient (100% EtOH, 95% EtOH, 70% EtOH) followed by epitope retrieval using a microwave at 2 × 5 min in target retrieval buffer, pH 9 (Agilent Technologies). Slides were subjected to *in situ* PLA according to manufacturer's instructions (Olink, Uppsala, Sweden). In brief, sections were blocked in Duolink blocking buffer, before incubation with primary antibodies overnight. After washing, appropriate PLUS and MINUS probes were applied, and ligation, rolling circle amplification, and detection with fluorescent probes were performed. Upon completion of the *in situ* PLA protocol, cells were counterstained using a DyLight650‐conjugated CD34 antibody and Hoechst 33342, before mounting with Fluoromount mounting medium (Merck). As technical control for each experiment, the same procedure was performed with the omission of either of the primary antibodies.

### Image acquisition and annotation

Images of the discovery TMA were acquired using a Leica confocal microscope SP8 with the Leica Application Suite X software (Leica Microsystem, Ketzlar, Germany) using HC PL CS2 20x and 40x objectives with 0.75 NA and 1.3 NA, respectively. The lasers used were diode 405, OPSL 488, OPSL 552, and diode 638 with 1 AU pinhole. The xy pixel size was 0.568 μm and 0.388 μm for the 20x and 40x objectives, respectively. Multispectral images of the validation TMA were acquired using the Vectra Polaris Automated Quantitative Pathology Imaging System automated scanning system (PerkinElmer, Waltham, MA, USA) using a 20x PL‐APO objective with NA 0.45. The gain, offset, and exposure time for each fluorophore were kept the same for all biopsies.

Processing of images and automated analysis was performed using the Cell Profiler software 27. All images of an experiment were processed equally. In order to improve visualization of PLA complexes, fluorescent punctuates were enhanced using the EnhanceOrSupress feature. PLA complexes were labeled as *trans* or *cis* based on their position relative to the CD34 positive blood vessel. *Cis* was defined as complexes localized within the CD34‐positive area, whereas *trans* was defined as complexes adjacent but not overlapping with the CD34 staining (maximum one nucleus away), as described previously 22. Complexes in *cis* and *trans* were scored as either present or absent. NRP1 IF staining was scored within three compartments; in endothelial cells (overlapping with CD34), in perivascular tumor cells (tumor cells adjacent to CD34 positive vessels), and general tumor cell expression. Each compartment was scored negative or positive for NRP1 expression. All scoring was performed independently by two investigators (EM and ES), blinded with regard to clinicopathological characteristics and outcome.

### Statistical analysis

For comparison of mean values, an unpaired two‐tailed Student's *t*‐test was performed as indicated and *p‐values* < 0.05 were considered statistically significant. Association of NRP1 levels with clinicopathological parameters was analyzed with Fisher's exact test or Pearson's chi‐square test. Probabilities of survival were estimated using the Kaplan–Meier method and log‐rank test. The correlation of patient survival time with VEGFR2/NRP1 *trans* complexes, NRP1 levels in tumor cells or NRP1 levels in perivascular tumor cells was evaluated using Cox proportional hazards regression model in uni‐ and multivariable analyses. The SPSS software package 21.0 (IBM Corporation, Armonk, NY, USA) was used for statistical analysis, and *p‐values* < 0.05 were considered statistically significant.

## Results

### Elevated NRP1 levels and VEGFR2/NRP1 complex formation in RCC

**Figure 1:**
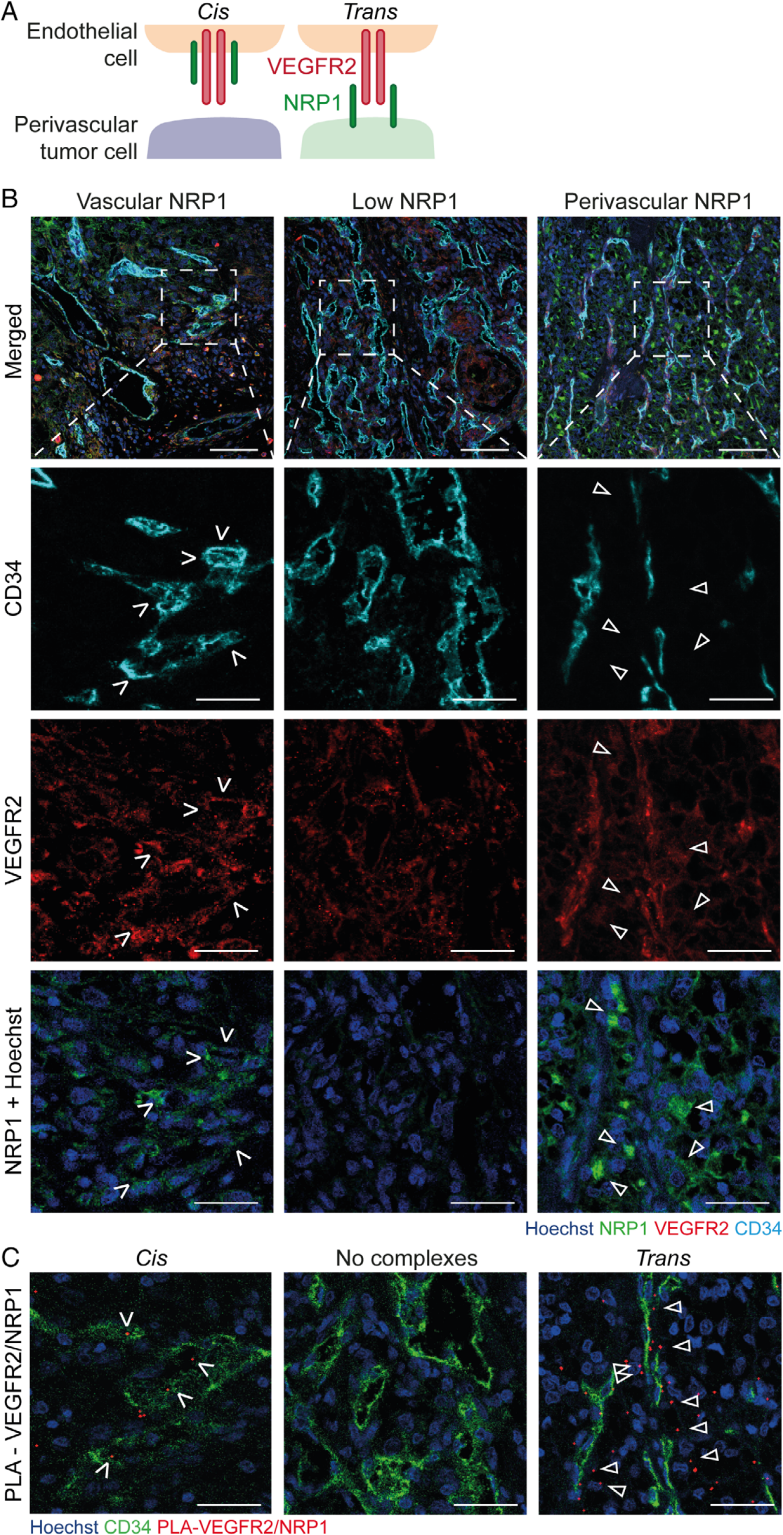
NRP1 expression correlates with VEGFR2/NRP1 complex formation. (A) Schematic figure of endothelial expressed VEGFR2 (red) and NRP1 (green) expressed either on endothelial or perivascular tumor cells, forming VEGFR2/NRP1 complexes in *cis* (left panel) and *trans* configurations (right panel). (B) Representative images of RCC patient biopsies immunostained for VEGFR2 (red), CD34 (cyan), and NRP1 (green) and counterstained using Hoechst33342 (blue). Top row shows an overview of merged immunostaining; boxed regions are shown as magnified individual channels below. Left column shows examples of NRP1 expressed by endothelial cells (white open arrows); middle column shows a tumor sample with low NRP1 expression. Right column shows NRP1‐positive perivascular tumor cells (white arrowheads). Scale bars, 100 μm (top row) and 40 μm. (C) *In situ* PLA for VEGFR2/NRP1 complex formation on sections consecutive to (B), highlighting the relationship between NRP1 expression pattern and complex formation in *cis* and *trans*. VEGFR2/NRP1 complexes are detected as red punctuates. Blood vessels were stained for CD34 (green) and nuclei counterstained using Hoechst33342 (blue). Left column shows *cis* complexes, localized within the endothelium (white open arrows). Middle column shows a tumor lacking VEGFR2/NRP1 complexes. Right column shows *trans* complexes, located adjacent to the endothelium (white arrowheads). Scale bars, 40 μm.

Formation of VEGFR2/NRP1 complexes in *trans* requires expression of VEGFR2 on endothelial cells and NRP1 by perivascular tumor cells (Figure 1A). To investigate the range of *NRP1* expression in human cancer, an exploratory screen was performed using publicly available TCGA datasets from 12 solid cancers and their corresponding normal tissues. The kidney clear cell carcinoma (KIRC) dataset showed approximately 10‐ to 50‐fold elevated levels of *NRP1* mRNA, as compared to normal kidney tissue (see supplementary material, Figure S1). This degree of *NRP1* upregulation was unique to KIRC. In the other cancer types, upregulation of NRP1 was modest or not observed (see supplementary material, Figure S1).

To identify the NRP1‐expressing cell type in RCC, IF staining was performed on a tissue microarray (TMA) consisting of samples from 64 RCC patients (referred to as the discovery cohort; see materials and methods for details). NRP1 protein expression was detected mainly in cancer cells but also in endothelial cells. NRP1 expression was scored in three compartments: (1) generally in tumor cells, (2) in perivascular tumor cells (arrows in Figure 1B), and (3) in the vessels (open arrows in Figure 1B). Of the 64 RCC patients in the discovery cohort, samples from 63 patients yielded informative staining of which 47 (75%) were positive for tumor cell‐NRP1, 45 (72%) for perivascular NRP1 and 22 (35%) for endothelial cell expressed NRP1.

Co‐staining for VEGFR2 and the vessel marker CD34 in RCC samples showed VEGFR2 expression by CD34‐positive vessels (Figure 1B and see supplementary material, Figure S2). The specificity and sensitivity of the antibodies were carefully validated (see materials and methods and supplementary material, Figure S3A,B). The pattern of VEGFR2 and NRP1 expression in RCC, that is, VEGFR2 in the endothelial cells and NRP1 in both endothelial and tumor cells, indicated that VEGFR2/NRP1 complexes could form both in *trans* and *cis* configurations. To identify such complexes in human RCC tissue, *in situ* PLA was performed on consecutive sections with antibodies against VEGFR2 and NRP1, as described previously (Figure 1C) 22. Patient biopsies were scored positive or negative for VEGFR2/NRP1 complexes in *trans* and *cis* configurations (see material and methods for details). Of the 63 patients analyzed, 47 (75%) displayed *trans* complexes and 17 (27%) displayed *cis* complexes. As technical control for each experiment, the same procedure was performed with the omission of either of the primary antibodies (see supplementary material, Figure S3C,D).

In summary, *NRP1* mRNA and NRP1 protein were highly expressed in RCC, in particular in perivascular tumor cells, allowing formation of *trans*‐complexes with VEGFR2 expressed in the endothelium.

### VEGFR2/NRP1 complexes in *trans* associate with reduced vessel area and size

**Figure 2:**
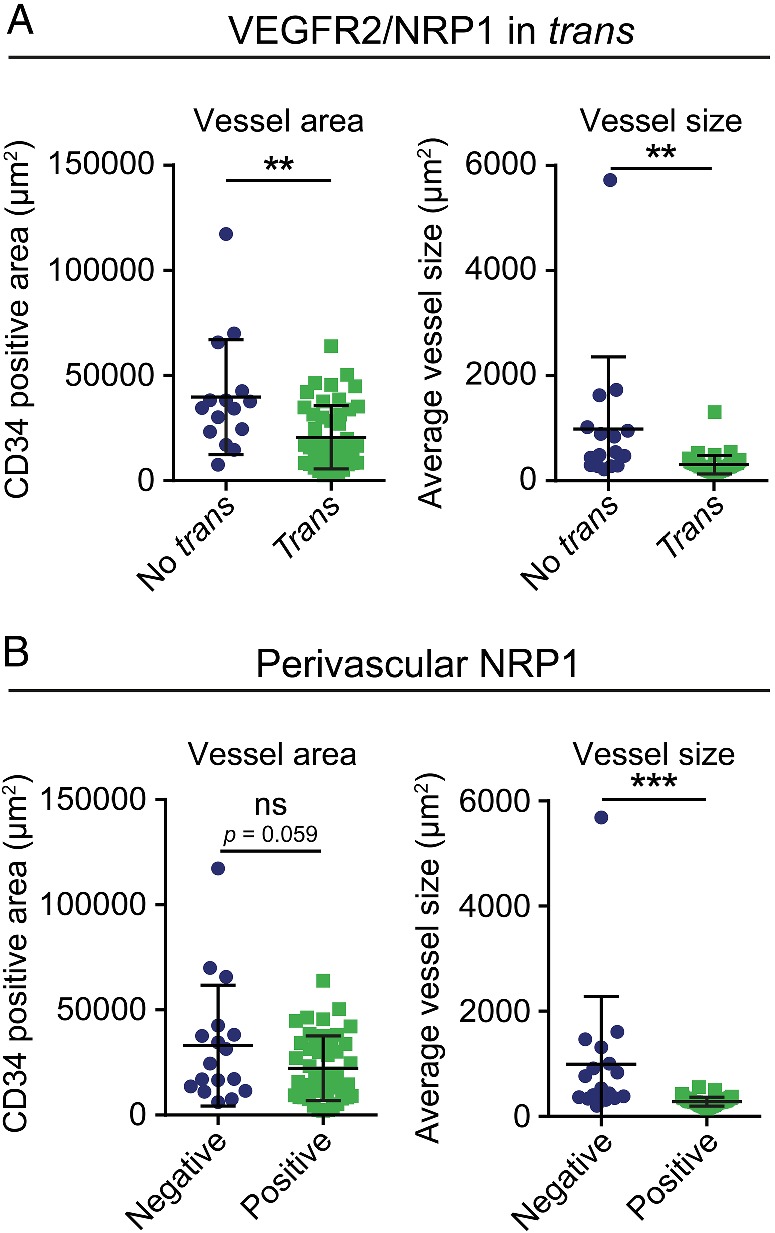
Perivascular NRP1 acting in *trans* is associated with reduced vessel area and size. (A) Analysis of total vascular area and size of individual vessels in patients scored negative (No *trans*, blue) or positive (*trans*, green) for VEGFR2/NRP1 *trans* complexes as determined by *in situ* PLA. Data are presented as mean values, ± SD *n* = 16 (No *trans*), *n* = 47 (*trans*), statistical analysis using Student's *t*‐test, ** = *p* < 0.01. (B) Analysis of total vascular area and size of individual vessels in patients scored negative (blue) or positive (green) for NRP1 expression in perivascular tumor cells based on IF staining. Data are presented as mean values, ± SD *n* = 18 (negative), *n* = 45 (positive), statistical analysis using Student's *t*‐test, *** = *p* < 0.005.

We have shown recently that the presence of VEGFR2/NRP1 *trans* complexes correlates with altered vessel parameters, including reduced total vessel area and vessel size in pancreatic adenocarcinoma (PDAC) 22. The characteristics of CD34‐positive vessels were therefore explored in the discovery cohort. In concordance with results from the previous study, vessel area and size were significantly smaller in RCC samples that were positive for VEGFR2/NRP1 complexes in *trans*, as compared to samples lacking *trans* complexes (Figure 2A).

To determine if the level of *trans* complexes (identified by PLA) was associated with general NRP1 expression in tumor cells (identified by IF staining), and in particular with expression in perivascular tumor cells, chi‐square tests were performed. There was a significant association between the presence of *trans* complexes and NRP1 expression in perivascular tumor cells (*p*‐value = 0.004), and between *trans* complexes and general NRP1 expression in tumor cells (*p*‐value = 0.036). The marked association of *trans* complexes with perivascular NRP1 expression prompted the question whether NRP1 expression, based on IF staining, also associated with vessel characteristics. As shown in Figure 2B, samples positive for perivascular NRP1 showed a trend toward smaller vessel area (*p*‐value = 0.059) and a significantly smaller vessel size, compared to samples lacking NRP1 expression.

In summary, patients with high NRP1 expression in tumor cells and in particular in perivascular tumor cells, showed a preferential presence of *trans* complexes. Moreover, samples with VEGFR2/NRP1 *trans* complexes, detected by PLA, or alternatively, NRP1 expression on perivascular or tumor cells, detected by IF staining, exhibited decreased overall vessel area and reduced vessel size.

### NRP1 interaction with the endothelium correlates with improved RCC overall survival

To explore the clinical relevance of VEGFR2/NRP1 *trans* complexes in RCC and the associated vessel parameters, staining for NRP1, VEGFR2, and CD34 was performed on an independent validation cohort of 314 RCC patients (see material and methods for details). Similar to the discovery cohort, patients were scored for general tumor cell‐, perivascular‐, and endothelial NRP1 expression. Among the 314 patients included in the TMA, 297 yielded informative staining; 224 (75%) were positive for tumor cell‐NRP1, 208 (70%) for perivascular NRP1, and 93 (31%) for endothelial cell‐NRP1.

**Figure 3:**
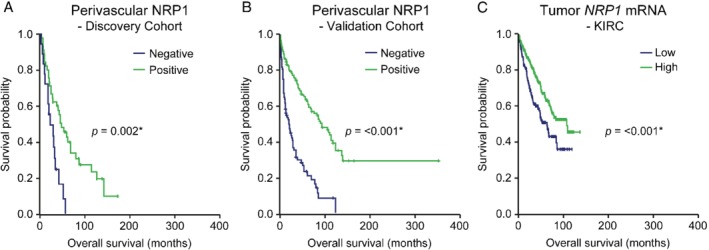
Perivascular NRP1 expression correlates to improved overall RCC survival. (A) and (B) Kaplan–Meier curves of overall survival in RCC patients negative (blue line, A *n* = 18, B *n* = 89) or positive (green line, A n = 45, B n = 208) for perivascular tumor cell expression of NRP1 in the discovery (A) and validation (B) cohorts. Statistical analysis using log‐rank test, *p*‐value <0.05 was considered significant, indicated by *. (C) Kaplan–Meier curve of overall survival in ccRCC patients with low or high levels of *NRP1* mRNA in the *KIRC* gene expression dataset (TCGA). NRP1 status is defined based on gene expression levels (see material and methods for cut‐off definition). Patients with low NRP1 expression (*n* = 133; blue), and high NRP1 expression (*n* = 400: green). Statistical analysis using log‐rank test, *p*‐value <0.05 was considered significant, indicated by *.

Next, perivascular NRP1 expression in the discovery and validation cohorts was analyzed for association with clinicopathological characteristics and patient survival. There was no association between positive NRP1 staining and relevant RCC clinicopathological characteristics including sex, age, histology, MSKCC‐score, tumor stage (T stage), and presence of metastasis at diagnosis (M stage) in the discovery cohort (Table 1). In the validation cohort, perivascular NRP1 was associated significantly with less metastasis, lower T stage and Fuhrman grade, and with clear cell histology (Table 1). Kaplan–Meier analyses (Figure 3A,B) showed that perivascular NRP1 expression correlated with a significantly better overall survival in both cohorts (discovery cohort; Log rank test, *p*‐value = 0.002, validation cohort; Log rank test, *p*‐value <0.001).

General tumor cell NRP1 expression was similarly analyzed with respect to clinicopathological characteristics and outcome in both RCC cohorts. In the discovery cohort, general expression of NRP1 in tumor cells was not associated with clinicopathological characteristics (see supplementary material, Table S2). In the validation cohort, general NRP1 expression associated with M stage, T stage, Fuhrman grade, and clear cell histology (see supplementary material, Table S2). General tumor cell expression also correlated with improved prognosis, both in the discovery (see supplementary material, Figure S4A, Log rank test, *p*‐value = 0.029) and validation cohort (see supplementary material, Figure S4B, Log rank test, *p*‐value <0.001).

To explore the impact of NRP1 in an additional independent RCC patient cohort, *NRP1* mRNA expression was combined with clinicopathological characteristics and survival data in the KIRC dataset for ccRCC. The lowest quartile of the cohort was used to identify patients with low *NRP1* expression and the top 75% was considered as patients with high *NRP1* expression. In the KIRC cohort, high *NRP1* mRNA expression was significantly associated with low T stage and lack of metastatic burden (see supplementary material, Table S2). Kaplan–Meier analysis demonstrated significantly improved overall survival for patients with high *NRP1* transcript levels (Figure 3C, Log rank test, *p*‐value <0.001).

NRP1 expression in endothelial cells also allowed complexes to form in *cis* with endothelial‐expressed VEGFR2 (Figure 1A,B). Therefore, survival analyses were performed in the discovery and validation cohorts after sub‐dividing patients into the following four groups: (1) NRP1 perivascular expression only, (2) endothelial expression only, (3) expression on both perivascular tumor and endothelial cells, and (4) no NRP1 expression. RCC patients scored negative for NRP1 expression, or with NRP1 expression only in endothelial cells (groups 2 and 4) displayed worse outcome as compared to patients with perivascular NRP1 expression (group 1) (see supplementary material, Figure S4C, Log rank test, *p*‐value = 0.015 and 0.001, respectively). Similar observations were made in the validation cohort (see supplementary material, Figure S4D, Log rank test, *p*‐value <0.001 and < 0.001, respectively). This observation of worse overall survival for patients with NRP1 expression in the tumor vessels is supported by previous findings that VEGFR2/NRP1 *cis*‐complexes promote tumor angiogenesis in mouse models 21. Of note, patients displaying both perivascular and endothelial expression of NRP1 did not differ significantly from patients with perivascular‐only expression in either of the two cohorts (see supplementary material, Figure 4C,D). These results indicate that the negative effect of the VEGFR2/NRP1 *trans* configuration dominates the positive *cis* effects in regulating angiogenesis, in congruence with mechanistic data from murine tumor models 21.

**Figure 4:**
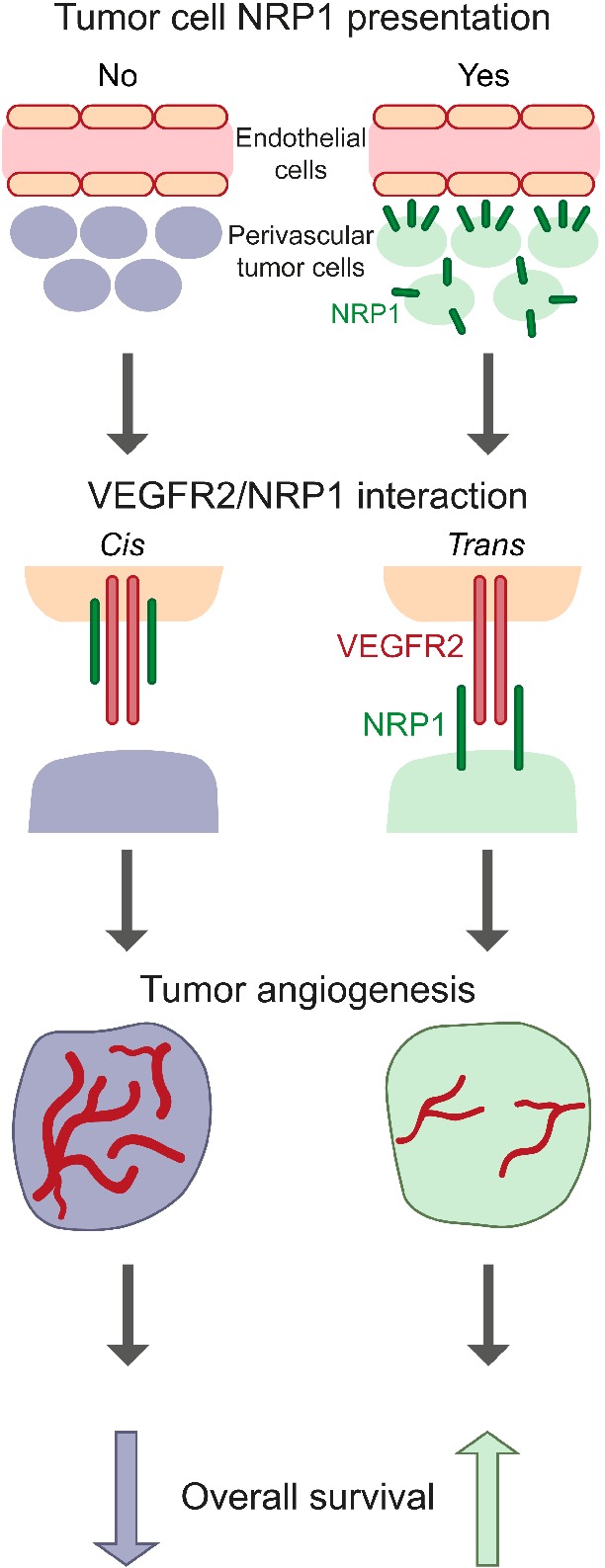
Presence of perivascular NRP1 allows VEGFR2/NRP1 *trans* complex formation, reducing tumor angiogenesis and enhancing patient survival. Schematic illustration showing the impact of endothelial cell NRP1 presentation (left column) compared to tumor cell presentation of NRP1 to endothelial VEGFR2 (right column). Presence of NRP1 in endothelial cells or perivascular tumor cells allows complex formation in *cis* or *trans*, respectively. *Cis* VEGFR2/NRP1 complex formation leads to increased vessel area and size (left column). In contrast, *trans* VEGFR2/NRP1 complex formation leads to reduced vessel parameters (right column), and improved overall survival in RCC patients.

In conclusion, NRP1 expression in the tumor cell compartment correlated with improved survival in two independent RCC cohorts, which was further supported by results from the KIRC ccRCC dataset. A more in‐depth analysis of the expression pattern underscored the clinical relevance of the interaction of perivascular NRP1 with endothelial VEGFR2.

### Perivascular NRP1 is an independent biomarker of RCC overall survival

To explore whether NRP1 is an independent marker of patient survival, univariable Cox‐regression analyses were performed. NRP1 expression in tumor cells in the discovery cohort showed a decreased risk of cancer‐related death (hazard ratio [HR] = 0.5, 95% confidence interval [CI] = 0.3–0.9, *p*‐value = 0.03). Similar results were shown for NRP1 expression in perivascular tumor cells (HR = 0.4, 95% CI = 0.2–0.7, *p*‐value = 0.003). In addition, for the validation cohort, there was decreased risk of cancer‐related death with NRP1 expression in tumor cells (HR = 0.3, 95% CI = 0.2–0.5, *p*‐value <0.001) and perivascular NRP1 (HR = 0.3, 95% CI = 0.2–0.4, *p*‐value <0.001). Multivariable analysis was performed on both cohorts including perivascular (Table 2) or general tumor cell expression of NRP1 (see supplementary material, Table S3) together with sex, age, histology, MSKCC‐score, Fuhrman grade, T stage, and M stage. The multivariable analyses identified independent favorable prognostic significance of expression of NRP1 in perivascular tumor cells in the discovery cohort (HR = 0.3, 95% CI = 0.1–0.6, *p*‐value <0.001), and in the validation cohort (HR = 0.5, 95% CI = 0.3–0.9, *p*‐value = 0.02) (Table 2), but not for total tumor cell expression of NRP1 (see supplementary material, Table S3). Multivariable analysis including clinicopathological characteristics was also performed on the *KIRC* gene expression dataset, showing independent prognostic significance for high *NRP1* transcript expression (HR = 0.6, 95% CI = 0.4–1.0, *p*‐value = 0.04) (see supplementary material, Table S3).

**Table 2:** Multivariable analysis of overall survival, including perivascular NRP1, in the discovery and validation cohorts

	Discovery Cohort			Validation Cohort		
	HR	95% CI	*p*‐value	HR	95% CI	*p*‐value
**Sex**						
Female	1		0.6	1		0.4
Male	1.3	0.6–2.9		0.8	0.5–1.4	
**Age**						
<60	1		0.08	1		0.003*
≥60	0.5	0.2–1.1		2.449	1.4–4.4	
**Histology**						
Non clear cell	1		0.1	1		<0.001*
Clear cell	2.5	0.7–8.9		0.24	0.1–0.5	
**MSKCC score**						
Low	1					
Intermediate	2.5	1.2–5.3	0.02*			
High	8.0	1.9–33.0	0.004*			
**Fuhrman grade**						
1				1		
2				1.3	0.7–2.6	0.4
3				1.1	0.5–2.5	0.7
4				2.2	0.9–5.8	0.1
**T stage**						
1	1			1		
2	0.5	0.2–1.6	0.3	1.2	0.4–3.8	0.7
3	2.6	1.0–6.9	0.05	1.1	0.3–3.6	0.9
4	0.8	0.1–7.6	0.8	2.5	0.8–7.8	0.1
**M stage**						
0	1		0.06	1		<0.001*
1	2.0	1.0–4.3		5.5	2.8–10.5	
**Perivascular NRP1**						
No	1		<0.001*	1		0.02*
Yes	0.3	0.1–0.6		0.5	0.3–0.9	

Abbreviations: CI, confidence interval; HR, hazard ratio; M stage, presence of distant metastasis; MSKCC score, Memorial Sloan‐Kettering Cancer Center score; NRP1, Neuropilin 1; T stage, size or direct extent of the primary tumor.

* Denotes statistical difference (*p* < 0.05).

Together, these data identified NRP1 expression in perivascular tumor cells as a novel independent marker for RCC patient prognosis.

## Discussion

The current study reveals novel findings of clinical relevance with regard to compartmentalized NRP1 expression in human RCC. The prognostic impact of NRP1 has been described earlier as being either unfavorable 28, 29, 30 or favorable 31 depending on the cancer type. However, the compartment‐specific expression of NRP1, and the ability to form VEGFR2/NRP1 complexes in *trans* were not explored in these studies. Findings from our previous work, supported by the present study, suggest that the NRP1 expression pattern, not only the overall expression levels, is of critical importance for tumor progression and patient prognosis 22. Thus, tumor cell expression of NRP1 (see supplementary material, Figure S4A,B) and more importantly, perivascular tumor cell expression (Figure 3A,B), correlated with improved outcome in two independent RCC cohorts. The underlying mechanisms of the favorable effect of perivascular NRP1 expression is the capacity to form *trans* complexes with VEGFR2, preventing VEGFR2 internalization and productive downstream signaling (Figure 4) 21. Thereby, tumor angiogenesis is suppressed, which in turn results in decreased tumor cell proliferation 21, 22. Arresting VEGFR2 on the cell surface through *trans* complex formation with NRP1 may also enhance efficient targeting of VEGFR2 using anti‐angiogenic therapy through slower turn‐over of the receptor; this scenario warrants further testing in mechanistic studies involving murine models of RCC. Furthermore, these data add to recent literature demonstrating prognostic impact of novel vascular and perivascular markers in RCC 32, 33, 34.

In contrast, endothelial expression of NRP1 was associated with a slightly worse prognosis (see supplementary material, Figure 4C,D). This is in line with a recent report that identified melanoma cell‐adhesion molecule (MCAM)/CD146 expression in tumor vessels as a prognostic marker for poor outcome in RCC. MCAM/CD146 is specifically expressed in the vasculature of ccRCC where it associates with VEGFR2 independently of VEGF 32.

Here, *in situ* detection of complex formation between VEGFR2 and NRP1 was performed using antibody‐mediated proximity ligation. To adapt our findings to a clinical setting, compartment‐specific NRP1 expression analysis was done using IF staining. It is notable that patients who had tumors that were positive for perivascular tumor cell expression of NRP1 also exhibited less‐vascularized tumors and improved overall survival. Results were further validated by analysis of *NRP1* transcripts in the KIRC ccRCC dataset. Patients with tumors that were positive for perivascular NRP1 expression in the validation cohort and high levels of *NRP1* mRNA in KIRC showed reduced metastatic spread. However, multivariable analyses identified independent favorable prognostic significance of expression of NRP1 only when specifically assessed in perivascular tumor cells and not for total tumor cell expression of NRP1. Moreover, a significant association between NRP1 status and metastasis could not be observed in the 64‐patient discovery cohort. A possible explanation is that the cohort was created to include only RCC patients who had metastasis at diagnosis or eventually developed metastatic disease. The small population size could also have affected the results.

In summary, we show for the first time that tumor cell–expressed NRP1 forms complexes with VEGFR2 expressed by the endothelium (*trans*) in human RCC tumors, and halts tumor angiogenesis, thereby improving patient survival. Perivascular NRP1 expression serves as a novel independent marker of improved survival. Future studies on larger population‐based RCC cohorts should be performed to confirm the application of NRP1 status as a clinical biomarker for survival, and further explore NRP1 as a predictive marker for anti‐angiogenic therapy. Our work also encourages additional studies to explore the compartment‐specific expression of biomarkers in tumors and the clinical relevance of receptor complexes in different configurations.

## Author contributions statement

EM, LCW, and ES conceived the project. EM and ES designed and performed the experiments. MJ, CL, LE, PS, and UH were responsible for tissue collection and the collection of clinical database information. EM, LCW, and ES wrote the manuscript. All authors reviewed and approved the final manuscript.

## Supporting information


**Supplementary figure legends**

**Figure S1.** Neuropilin 1 (*NRP1*) mRNA expression in solid tumors and normal tissue controls

**Figure S2.** Tumor NRP1 expression

**Figure S3.** Immunofluorescence (IF) isotype controls and *in situ* proximity ligation assay (PLA)‐negative controls

**Figure S4.** Correlation between overall survival and general tumor cell NRP1 expression or compartment specific expression of NRP1

**Table S1.** Primary and secondary antibodies for immunofluorescence (IF)
**Table S2.** Association of clinicopathological characteristics with tumor cell NRP1 expression status in the discovery and validation cohorts and *NRP1* mRNA expression level in the publicly available gene expression dataset of clear cell renal cell carcinoma, denoted KIRC.
**Table S3.** Multivariable analysis of overall survival in the discovery, validation, and KIRC cohorts

## Data Availability

The data that support the findings of this study are available from the corresponding author ES, upon reasonable request.
