# Supplementary material for: Perivascular Neuropilin‐1 expression is an independent marker of improved survival in renal cell carcinoma
Source: J Pathol. 2020 Jan 29;250(4):387–96. doi: 10.1002/path.5380 (PMC7155095; doi:10.1002/path.5380)
Supplement: Supplementary file 1 — Supplementary figure legends [file PATH-250-387-s006.docx]

**Perivascular Neuropilin-1 expression is an independent marker of improved survival in renal cell carcinoma**

**Morin *et al. J Pathol* DOI: 10.1002/path.5380**

**Supplementary figure legends**

**Figure S1.** *NRP1* mRNA expression in solid tumors and normal tissue controls

Comparison of *NRP1* expression in publicly available gene expression datasets of 12 solid cancer types (red) and normal control tissues (grey) presented as transcripts per million. Datasets used; TCGA(tumor), TCGA(normal) and GTEx. Bladder cancer n tumor(T)/normal(N) = 404/28, breast cancer n T/N = 1085/291, colon cancer n T/N = 275/349, glioblastoma n T/N = 163/207, clear-cell renal-cell carcinoma n T/N = 523/100, papillary renal-cell carcinoma n T/N = 286/60, liver cancer n T/N = 369/160, lung adenocarcinoma n T/N = 483/347, lung squamous-cell carcinoma n T/N = 486/338, pancreatic cancer n T/N = 179/171, prostate cancer n T/N = 492/152 and gastric cancer n T/N = 408/211. Log_2_FC = 1.0 and *p-*value <0.05 were set as cut-off for significant difference of NRP1 expression between tumor and control tissue, denoted by *.

**Figure S2.** Tumor NRP1 expression

Overview images representing tumors scored positive for endothelial NRP1 (left column), low expression of perivascular NRP1 (middle column) and high expression of perivascular NRP1 (right column). Upper row shows staining for VEGFR2 (red), NRP1 (green), CD34 (cyan) and counterstaining for nuclei using Hoechst33342 (blue). Second row shows staining for VEGFR2 (red), third row shows staining for CD34 (cyan) and bottom row shows staining for NRP1 (green). Scale bars 100 µm.

**Figure S3.** Immunofluorescence isotype controls and *in situ* PLA negative controls

(A) Representative immunofluorescence images of pelleted consecutive sectioned paraffin‐embedded porcine artic endothelial cell expressing VEGFR2 and NRP1 (PAE/VEGFR2/NRP1) stained with target specific antibodies (left column) or isotype control (right column) against NRP1 (green) and VEGFR2 (red) with Hoechst counterstaining to visualize nuclei. Top panels are merged images of the individual channels shown below. Scale bars 100 µm.

(B) Representative immunofluorescence images of consecutive RCC TMA sections stained with target specific antibodies (left column) or isotype control (right column) against NRP1 (green), VEGFR2 (red) and CD34 (teal) with Hoechst counterstaining to visualize nuclei. Top panels are merged images of the individual channels shown below. Scale bars 100 µm.

(C and D), technical controls of *in situ* PLA reaction performed on consecutive sections to confirm specificity (red dots). (C) *in situ* PLA reaction with only NRP1 antibody present (VEGFR2 antibody omitted). (D) *in situ* PLA reaction with only VEGFR2 antibody present (NRP1 antibody omitted). Scale bars 20 µm.

**Figure S4.** Correlation between overall survival and general tumor cell NRP1 expression or compartment specific expression of NRP1

(A and B), Kaplan–Meier curve showing overall survival of patients in the discovery (A) and validation (B) RCC cohorts, negative (blue line, A n=16, B n=73) or positive (green line, A n=47, B n= 224) for NRP1 protein in the tumor cells. Statistical analysis using log-rank test, *p*-value <0.05 was considered significant, indicated by *.

(C) and (D), Kaplan–Meier curve of overall survival of RCC patients in the in the discovery (C) and validation (D) cohort sub-divided according compartment-specific expression of NRP1. Patients scored positive for NRP1 exclusively in perivascular tumor cells (blue line; A n=30, B n=143), for NRP1 in both endothelial and perivascular tumor cells (green; n=15, B n=65), NRP1 only in endothelial cells (beige; A n=7, B n=28) and no NRP1 expression (purple; A n=11, B n=61). Dotted lines indicate groups with statistically significant (*) differences in survival (*p*-value <0.05).
