# Supplementary figures and images for: Perivascular Neuropilin‐1 expression is an independent marker of improved survival in renal cell carcinoma
Source: J Pathol. 2020 Jan 29;250(4):387–96. doi: 10.1002/path.5380 (PMC7155095; doi:10.1002/path.5380)

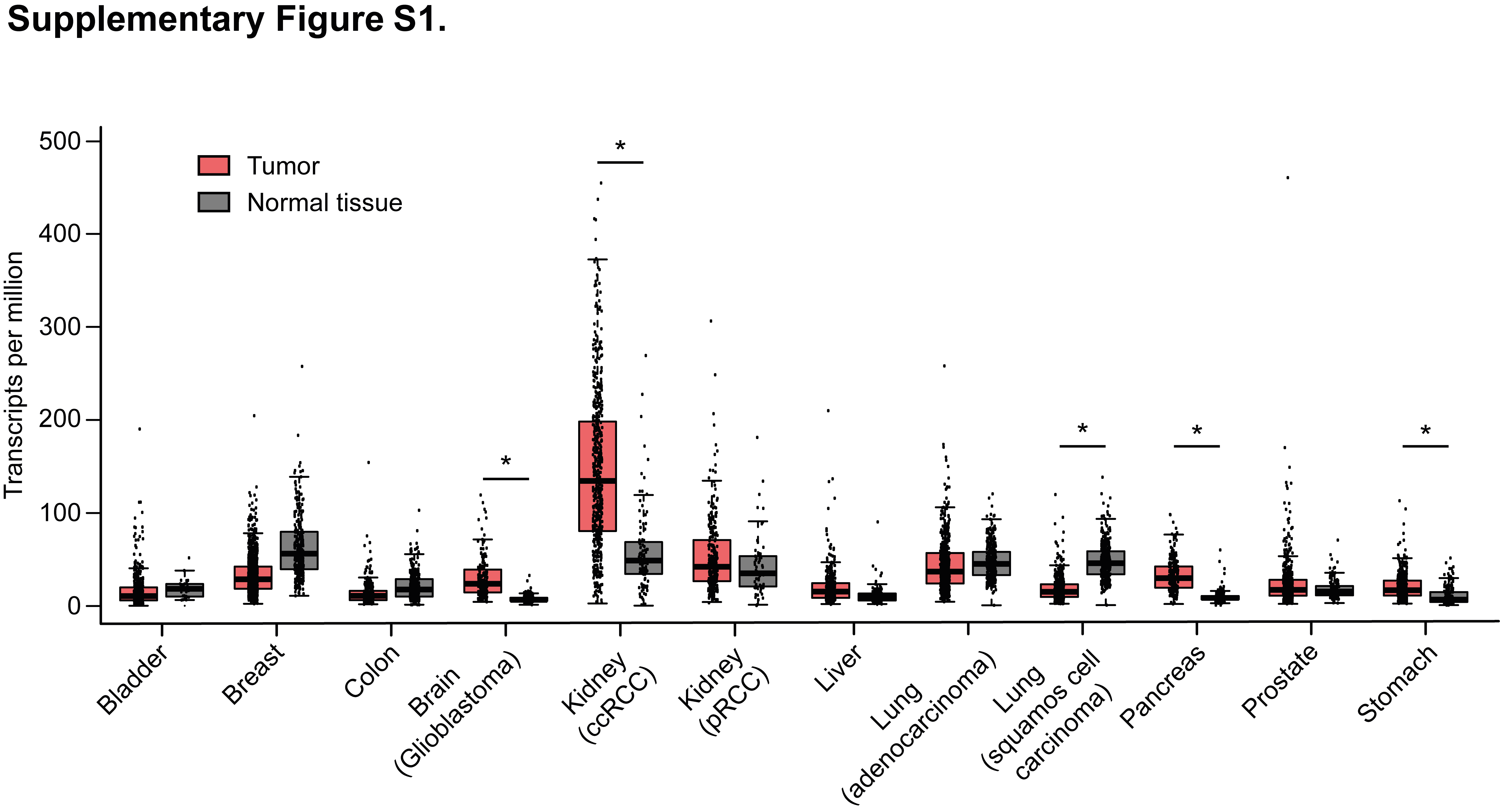

Supplement: Supplementary file 2 — Figure S1. Neuropilin 1 (NRP1) mRNA expression in solid tumors and normal tissue controls [file PATH-250-387-s001.tif]

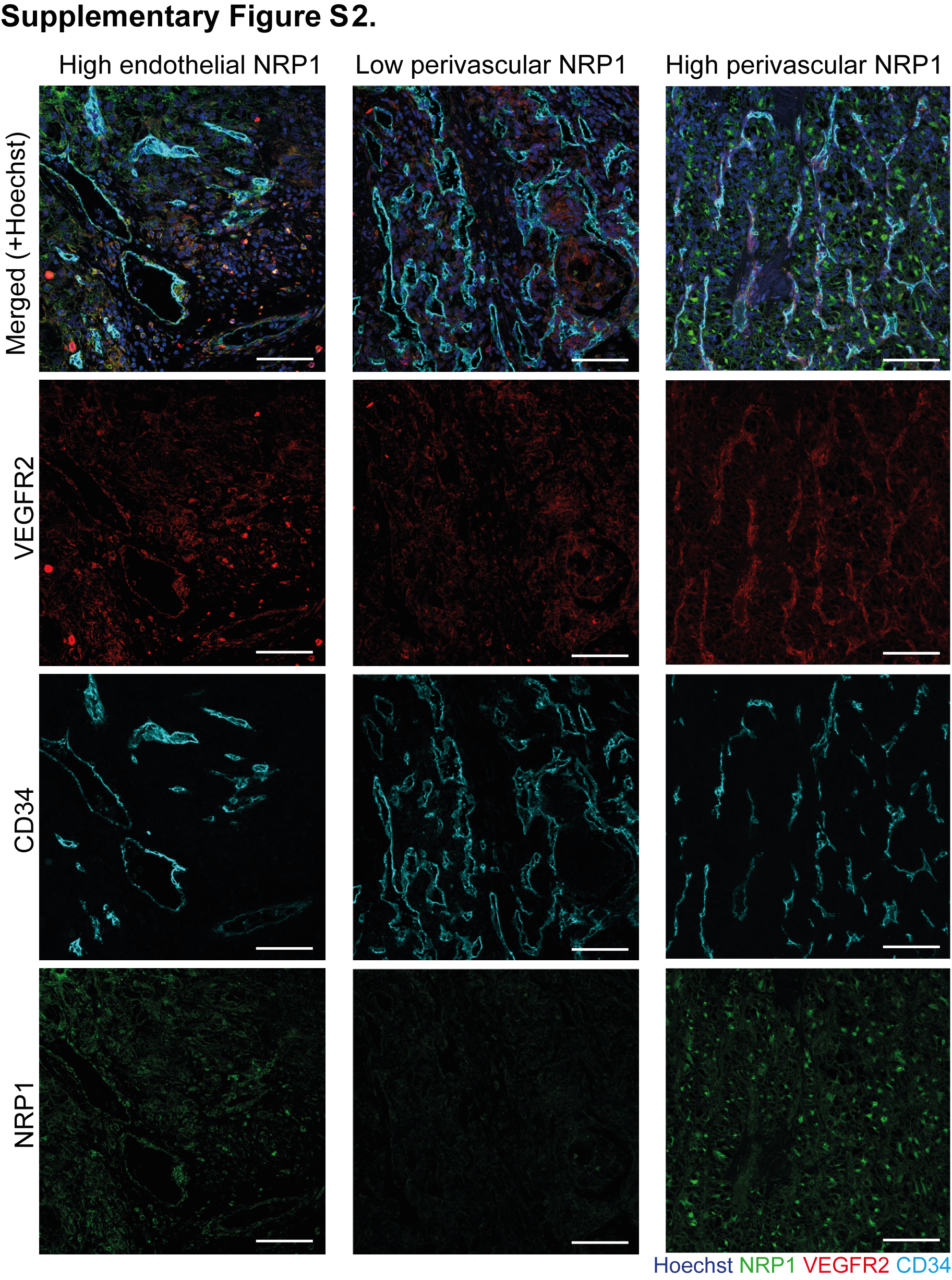

Supplement: Supplementary file 3 — Figure S2. Tumor NRP1 expression [file PATH-250-387-s002.tif]

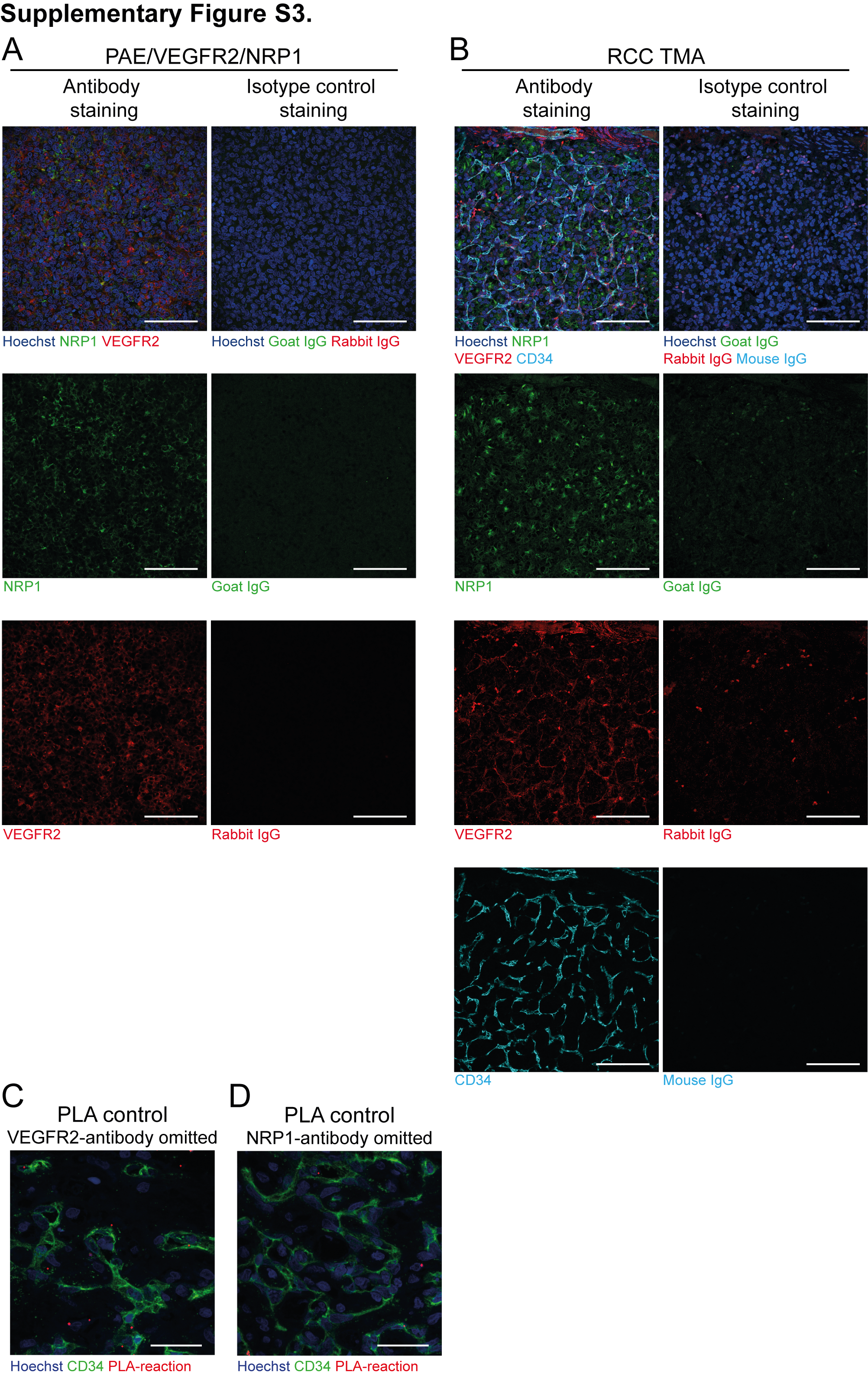

Supplement: Supplementary file 4 — Figure S3. Immunofluorescence (IF) isotype controls and in situ proximity ligation assay (PLA)‐negative controls [file PATH-250-387-s003.tif]

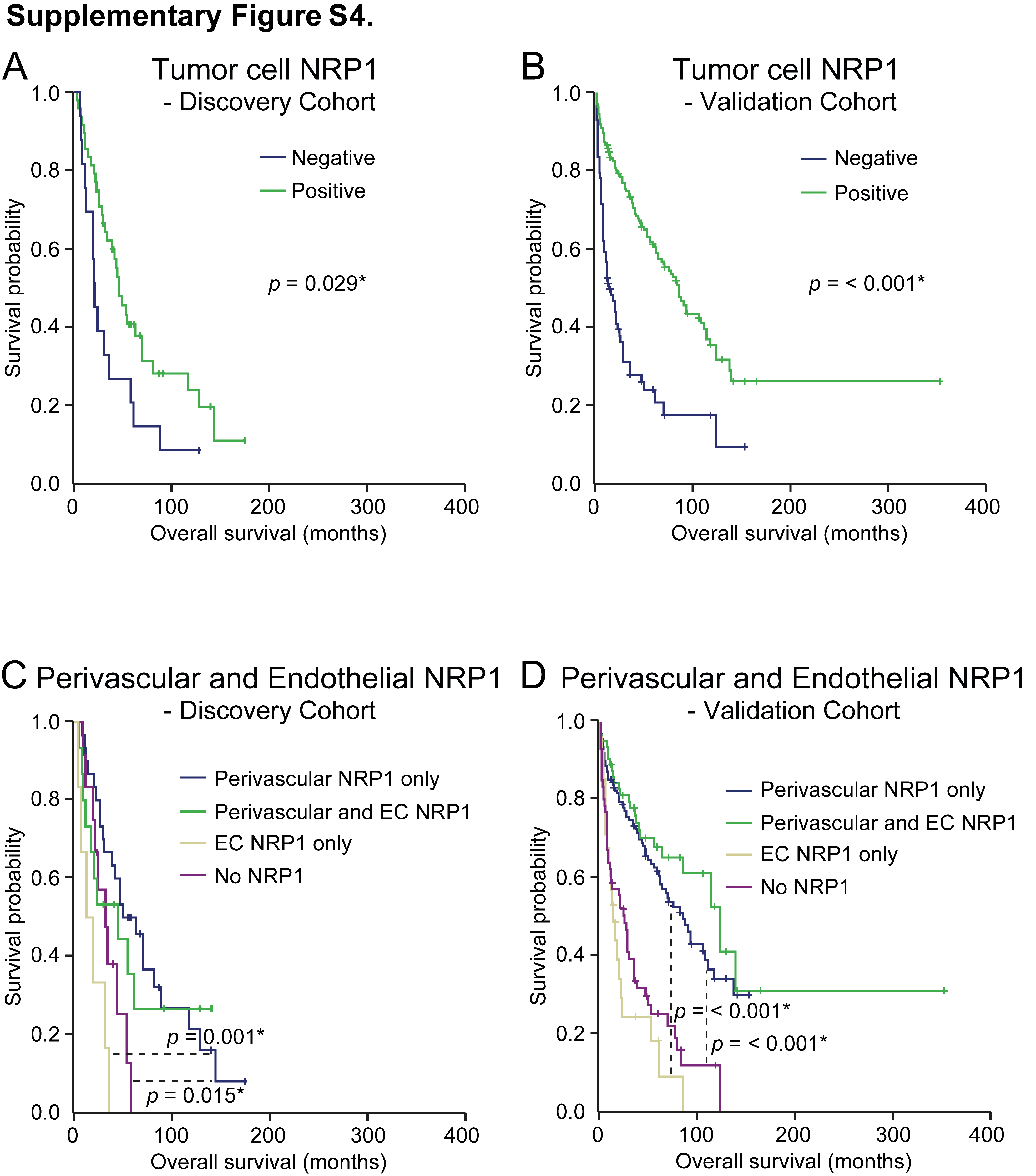

Supplement: Supplementary file 5 — Figure S4. Correlation between overall survival and general tumor cell NRP1 expression or compartment specific expression of NRP1 [file PATH-250-387-s004.tif]
