# Supplementary material for: Perivascular Neuropilin‐1 expression is an independent marker of improved survival in renal cell carcinoma
Source: J Pathol. 2020 Jan 29;250(4):387–96. doi: 10.1002/path.5380 (PMC7155095; doi:10.1002/path.5380)
Supplement: Supplementary file 6 — Table S1. Primary and secondary antibodies for immunofluorescence (IF) Table S2. Association of clinicopathological characteristics with tumor cell NRP1 expression status in the discovery and validation cohorts and NRP1 mRNA expression level in the publicly available gene expression dataset of clear cell renal cell carcinoma, denoted KIRC. Table S3. Multivariable analysis of overall survival in the discovery, validation, and KIRC cohorts [file PATH-250-387-s005.pdf]

Perivascular Neuropilin-1 expression is an independent marker of improved survival in renal cell carcinoma

Morin *et al.* *J Pathol* DOI: 10.1002/path.5380

Supplementary tables

Table S1. Primary and secondary antibodies for immunofluorescence (IF)

Application	Primary Antibodies	Secondary Antibodies
**IF Discovery Cohort**	Anti-VEGFR2, dilution 1:150 (2479, Cell Signaling Technology, Danvers, MA, USA)<br><br>Anti-NRP1, dilution 1:100 (AF566, R&D Systems, Minneapolis, MI, USA)<br><br>Anti-CD34, dilution 1:100 (IR63261-2, clone QBEnd10, Agilent Technologies, Santa Clara, CA, USA)	Alexa Fluor®555 donkey anti-rabbit, dilution 1:400 (A-31572, Invitrogen, Carlsbad, CA, USA)<br><br>Alexa Fluor®488 donkey anti-goat, dilution 1:400 (A-11055, Invitrogen)<br><br>Alexa Fluor®647 donkey anti-mouse, dilution 1:400 (A-31571, Invitrogen)
**IF Validation Cohort**	Anti-VEGFR2, dilution 1:150 (2479, Cell Signaling Technology)<br><br>Anti-NRP1, dilution 1:100 (AF566, R&D Systems)<br><br>Anti-CD34, dilution 1:100 (IR63261-2, clone QBEnd10, Agilent Technologies)	Alexa Fluor®555 donkey anti-rabbit, dilution 1:400 (A-31572, Invitrogen)<br><br>Alexa Fluor®647 donkey anti-goat, dilution 1:400 (705-605-147, Jackson ImmunoResearch, Philadelphia, PA, USA)<br><br>Alexa Fluor®488 donkey anti-mouse, dilution 1:400 (A-21202, Invitrogen)

Table S2. Association of clinicopathological characteristics with tumor cell NRP1 expression status in the discovery and validation cohorts and *NRP1* mRNA expression level in the KIRC cohort.

	Discovery Cohort				Validation Cohort				KIRC cohort (*NRP1* mRNA)			
	**Total** n=63	Negative n=16	Positive n=47	*p*-value	**Total** n=297	Negative n=73	Positive n=224	*p*-value	**Total** n=533	Low n=133	High n=400	*p*-value
**Sex**												
**Female**	14	3	11	1[1]	129	28	101	0.31[1]	188	50	138	0.518[2]
**Male**	49	13	36		168	45	123		345	83	262	
**Age**												
**<60**	12	3	9		98	18	80		245	64	181	
**≥60**	51	13	38	1[1]	196	54	142	0.08[1]	288	69	219	0.565[2]
**Missing**					3	1	2					
**Histology**												
**Non Clear Cell**	4	2	2		21	13	8					
**Clear Cell**	58	14	44	0.27[1]	236	45	191	<0.001[1]*				
**Missing**	1	0	1		40	15	25					
**MSKCC-score**												
**Low**	25	6	19									
**Intermediate**	31	7	24	0.92[2]								
**High**	3	1	2									
**Missing**	4	2	2									
**Fuhrman grade**												
**1**					113	13	100					
**2**					105	24	81					
**3**					54	22	32	<0.001[1]*				
**4**					21	13	8					
**Missing**					4	1	3					
**T-stage**												
**1**	10	1	9		33	4	29		273	57	216	
**2**	14	2	12		38	9	29		69	27	42	
**3**	37	13	24	0.23[2]	34	7	27	0.01[1]*	180	44	136	0.006[2]*
**4**	1	0	1		61	25	36		11	5	6	
**Missing**	1	0	1		131	28	103		0	0	0	
**N-stage**												
**0**									243	67	176	
**1**									16	8	8	0.055[2]
**Missing**									274	58	216	
**M-stage**												
**0**	39	8	31		239	45	194		433	93	340	
**1**	24	8	16	1[1]	58	28	30	<0.001[1]*	79	28	51	0.007[2]*
**Missing**	0	0	0						21	12	9	

Abbreviations: RCC = renal cell carcinoma; KIRC = kidney renal clear cell carcinoma; MSKCC-score = Memorial Sloan-Kettering Cancer Centre score; T-stage = size or direct extent of the primary tumor; N-stage = spread to regional lymph nodes; M-stage = presence of distant metastasis. NRP1 = Neuropilin 1.
Statistical analysis: [1]Fisher's exact test, [2]Pearson's chi-square. * Denotes statistical difference ($p < 0.05$).

Table S3. Multivariable analysis of overall survival in the discovery, validation, and KIRC cohorts.

	Discovery Cohort			Validation Cohort			KIRC Cohort		
	HR	95% CI	*p*-value	HR	95% CI	*p*-value	HR	95% CI	*p*-value
**Sex**									
**Female**	1		0.9	1		0.5	1		0.4
**Male**	0.9	0.4–2.1		0.8	0.5–1.4		1.2	0.8–1.9	
**Age**									
**<60**	1		0.1	1		0.01*	1		0.05
**≥60**	0.5	0.2–1.2		2.2	1.2–4.1		1.6	1.0–2.5	
**Histology**									
**Non Clear Cell**	1		0.2	1		<0.001*			
**Clear Cell**	2.3	0.6–8.7		0.2	0.1–0.5				
**MSKCC-score**									
**Low**	1								
**Intermediate**	0.9	1.1–5.1	0.03*						
**High**	1.9	1.7–26.5	0.007*						
**Fuhrman grade**									
**1**				1					
**2**				1.4	0.7–2.8	0.3			
**3**				1.4	0.7–3.1	0.4			
**4**				2.5	1–6.6	0.06			
**T-stage**									
**1**	1			1			1		
**2**	0.7	0.2–2.0	0.5	1.2	0.4–3.8	0.8	0.7	0.3–1.4	0.3
**3**	2.0	0.7–5.6	0.2	1.2	0.4–4	0.8	1.8	1.1–3.0	0.03*
**4**	0.7	0.1–6.4	0.7	2.9	0.9–8.9	0.06	1.2	0.3–4.0	0.8
**N-stage**									
**0**							1		0.05*
**1**							2.3	1.0–5.1	
**M-stage**									
**0**	1		0.1	1		<0.001*	1		<0.001*
**1**	1.8	0.8-3.8		5.1	2.7-9.7		3.0	1.8-5.0	
**TC NRP1 protein**									
**No**	1		0.3	1		0.3			
**Yes**	0.6	0.3-1.4		0.7	0.3-1.3				
***NRP1* mRNA**									
**Low**							1		0.04*
**High**							0.6	0.4-1.0	

Abbreviations: RCC = renal cell carcinoma; KIRC = kidney renal clear cell carcinoma; HR = hazard ratio; CI = confidence interval; MSKCC-score = Memorial Sloan-Kettering Cancer Centre score; T-stage = size or direct extent of the primary tumor; N-stage = spread to regional lymph nodes; M-stage = presence of distant metastasis; NRP1 = Neuropilin 1
* Denotes statistical difference (p <0.05).
